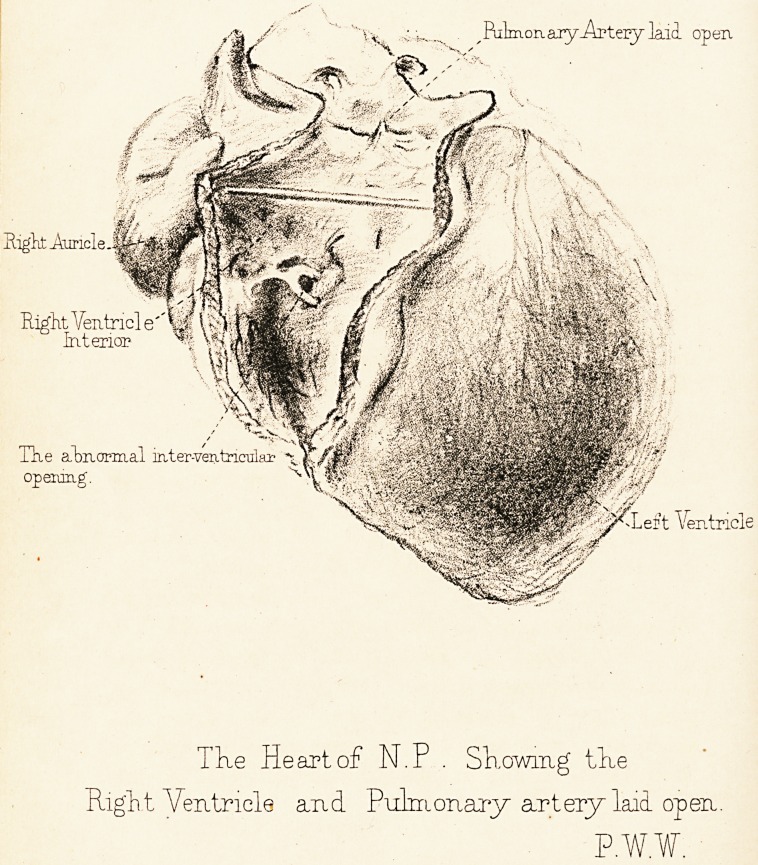# Notes on a Rare Case of Morbus Cæruleus

**Published:** 1888-09

**Authors:** P. Watson Williams

**Affiliations:** Assistant-Physician, Bristol Royal Infirmary


The Heart of N.P. Showing the
Right Ventricle and Pulmonary artery laid open.

P.W.W.

### Clinical Records.

NOTES ON A RARE CASE OF MORBUS CÆRULEUS. By P. WATSON WILLIAMS, M.B. Lond., Assistant-Physician, Bristol Royal Infirmary.

The accompanying drawing is that of a heart of a girl, N. P., aged 7, who died in consequence of the malformation depicted. In this drawing the right ventricle is shown laid open, the pulmonary artery being likewise slit up. There is no opening from this right ventricle into the auricle; but the abnormal communication between the ventricles is shown in the drawing.

In this heart the auricles form practically one cavity, and are very dilated. The patent foramen ovale is very large, and there could have been no obstruction to the passage of blood from the right to the left auricle.

The mitral orifice is unusually large, and its cusps thickened. There is no opening, nor any sign of the tricuspid valve between the right auricle and ventricle.

The pulmonary artery and aorta and their valves are normal. The ductus arteriosus was not patent.

The left ventricle is hypertrophied, its cavity large; the right ventricular cavity is small, and the walls very thin. At the upper or basal extremity of the ventricular septum is a circular portion, the size of a threepenny-piece, where the muscular substance is absent, the deficiency being replaced by fibrous membrane resembling a thickened valve, and having near its centre an opening which just admits a No. 8 catheter. Attached to the edges round the opening are some structures resembling minute chordæ tendineæ; there are also a few small bead-like fibrinous deposits on these edges. This interventricular communication is situated immediately below the interval between the left and posterior aortic valve segments. The muscular substance is well nourished, and the coronary arteries rather large. Probably the course of the blood-current was that the small amount of blood going to the lung by the pulmonary artery was returned to the right auricle, thence to the left auricle, to the left ventricle, and from the left ventricle part went to the right ventricle, through the abnormal aperture, the greater portion going to the aorta.

It is worthy of note that, in spite of this deformity in the heart, the child was bright and cheerful, and very intelligent. She was born at full time, and for some months after, the mother states, was only a little blue about the cheeks. During life a systolic bruit was audible over the whole of the præcordia, below the nipple level, most marked at the base of the xiphoid cartilage.

*Remarks.*—This heart falls under the class of cases malformed from *arrested development*, and this probably occurred about the tenth week of fœtal life: for the interventricular septum begins to form at the apex about the sixth week, and by the tenth would have nearly reached the base of the ventricles; the auricular septum begins to develop later than the ventricular septum, and this accounts for its rudimentary condition in the case. The arterial bulb, which divides into the aorta and pulmonary artery at the tenth week, has undergone complete division in this specimen; about the tenth week also the auriculo-ventricular valves begin to form by the growing in of fleshy plates from the walls of the common auriculo-ventricular cavities. Thus, if we allow that these plates became fused at that time, instead of developing into a tricuspid valve, the currents of blood of necessity passing between the auricles would maintain the patency of the foramen ovale.

When the ventricular septum is deficient, it is usually, as one would expect it to be, at the base of the septum, the last-formed portion, as in this case.

---

[The heart from which the drawing was made is preserved in the museum of the Bristol Royal Infirmary.]

## Figures and Tables

**Figure f1:**